# Supplementary material for: Pan-Cancer Genomic Scars of Alternative End Joining and Single-Strand Annealing
Source: bioRxiv. 2026 May 26:2026.05.22.727283. Preprint. [Version 1] doi: 10.64898/2026.05.22.727283 (PMC13232104; doi:10.64898/2026.05.22.727283)
Supplement: Supplement 1 [file media-1.pdf]

Supplementary Materials of "Pan-Cancer Genomic Scars of Alternative End Joining and Single-Strand Annealing"

Ashini Modi [1] , Alessandro Zito [2,3] , Giovanni Parmigiani [2,3]

[1] Harvard College, Harvard University, Cambridge, MA, USA.
[2] Department of Biostatistics, Harvard T.H. Chan School of Public Health, Boston, MA, USA
[3] Department of Data Science, Dana Farber Cancer Institute, Boston, MA, USA

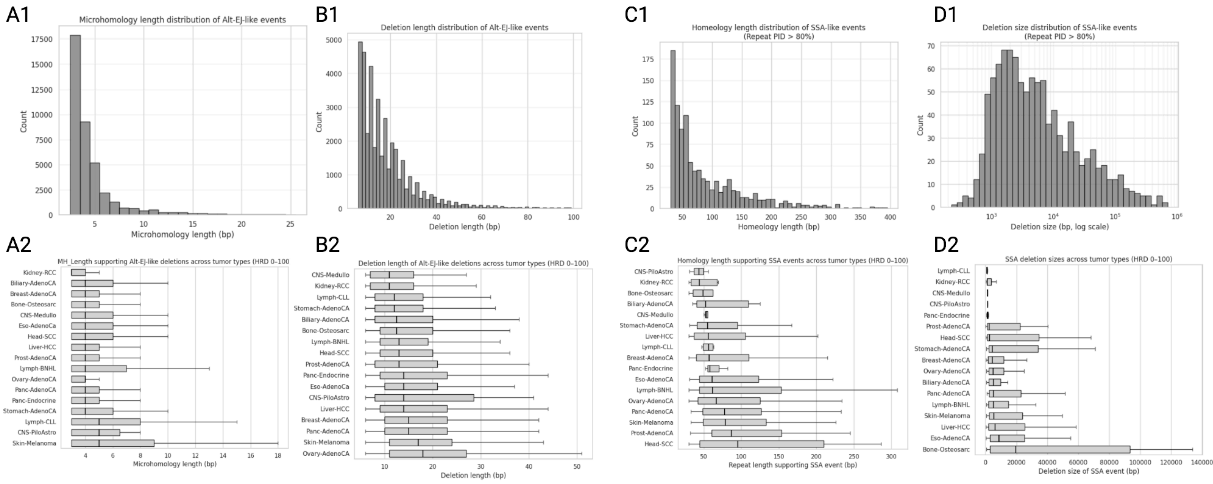

Fig. S1 | Homology length and deletion size characteristics of Alt-EJ– and SSA-like events.
a,b, Distribution of microhomology length **(a1)** and deletion length **(b1)** for Alt-EJ-like events across all tumors. **a2,b2,** Distribution of microhomology length **(a2)** and deletion length **(b2)** for Alt-EJ-like events for each tumor type. Box plots show median and interquartile range, with whiskers extending to 1.5× the interquartile range. **c,d,** Distribution of homeology length (repeat sequence identity >80%) **(c1)** and deletion size (log scale) **(d1)** for SSA-like events across all tumors. **c2,d2,** Distribution of homeology length **(c2)** and deletion size **(d2)** for SSA-like events for each tumor type.

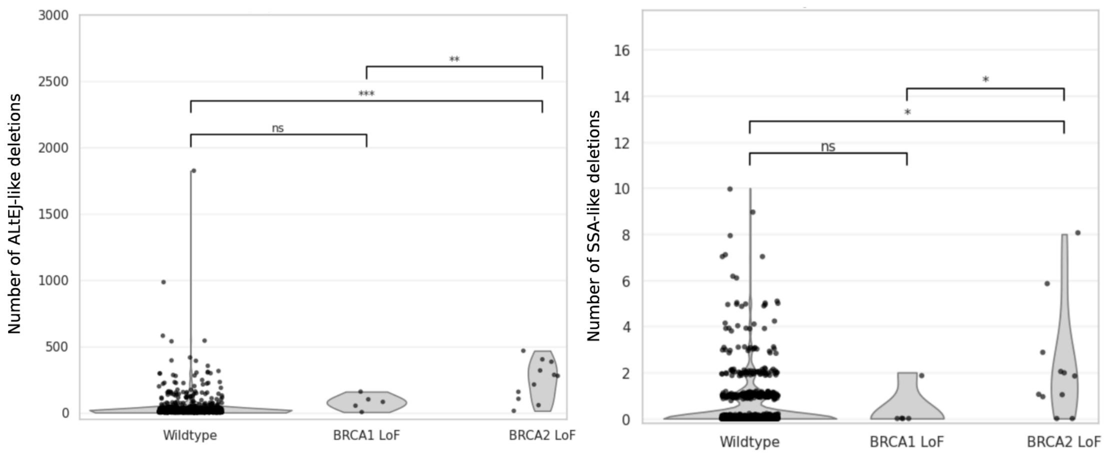

Fig. S2 | Increased Alt-EJ and SSA usage in BRCA2 but not BRCA1-deficient tumors.

a, Number of Alt-EJ-like deletions per tumor in wild-type, BRCA1 loss-of-function (LoF) and BRCA2 LoF groups. **b,** Number of SSA-like deletions per tumor across the same groups. Points represent individual tumors overlaid on violin plots showing the distribution within each group. Statistical significance between groups is indicated via Welch's t-tests (ns, not significant; *P < 0.05; **P < 0.01; ***P < 0.001). Variants were classified as LoF if they were frameshift, stop codon gain, splice variant, or start codon loss.

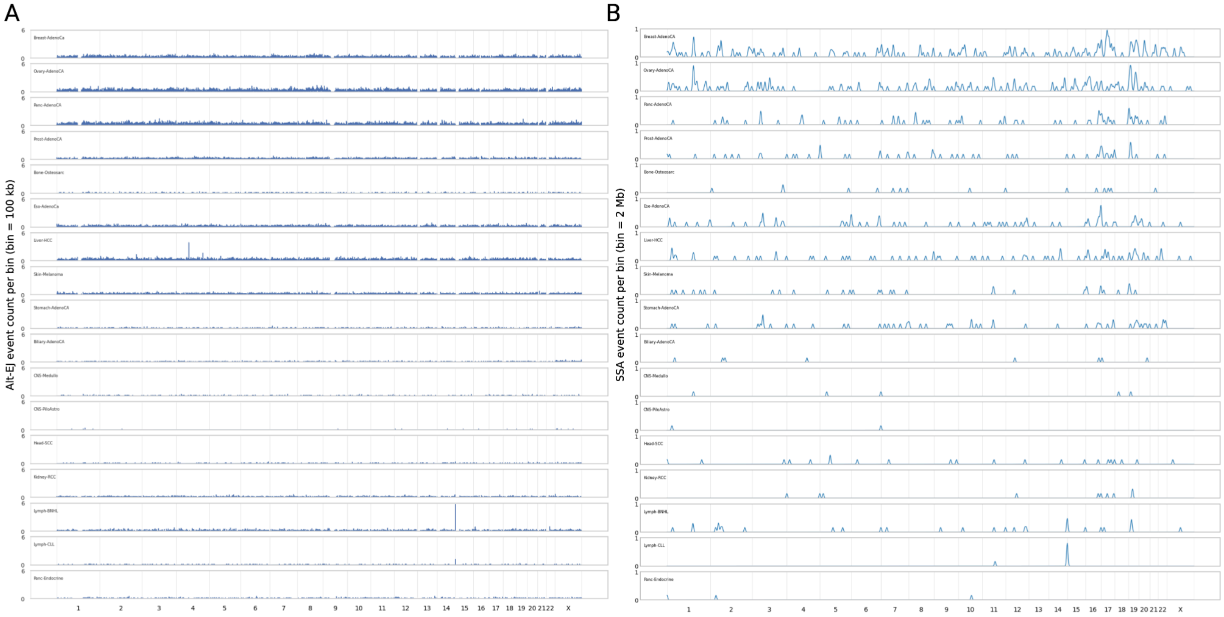

Fig. S3 | Genome-wide distribution of Alt-EJ- and SSA-like deletions across tumor types.
a, Genome-wide distribution of Alt-EJ-like deletions across individual tumor types, shown as event counts per 100 kb bin with chromosomes plotted sequentially along a continuous hg19 coordinate axis. Each row represents a tumor type. **b,** Genome-wide distribution of SSA-like deletions across the same tumor types, shown as event counts per 2 Mb bin. Each row represents a tumor type.

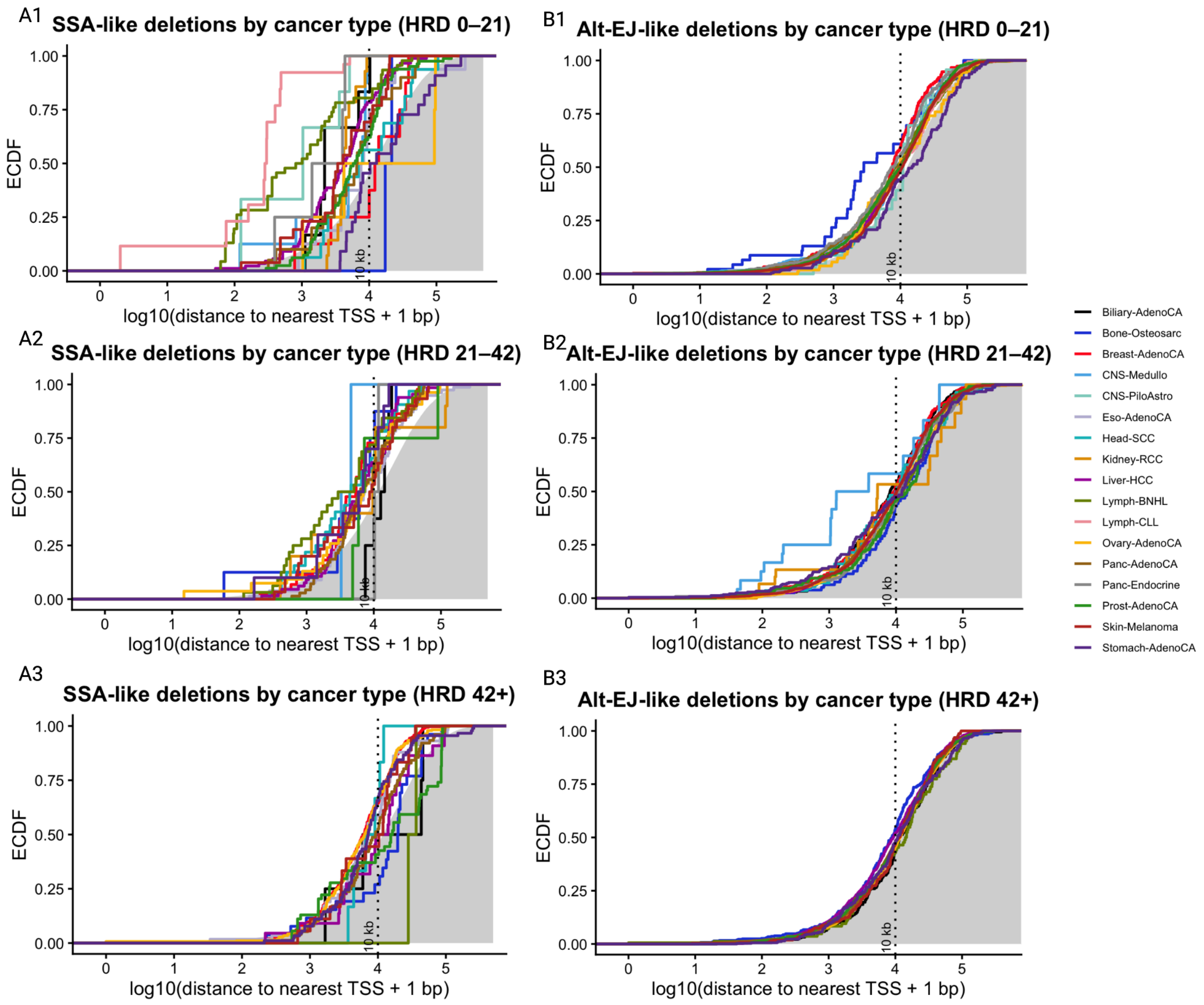

Fig. S4 | Distribution of SSA- and Alt-EJ-like deletion breakpoints relative to transcription start sites (TSS). **a1-a3**, Empirical cumulative distribution functions (ECDFs) of distances from SSA-like deletion breakpoints to the nearest TSS, shown for individual cancer types within (**a1)** HRD 0-21, **(a2)** HRD 21-42, and **(a3)** HRD >42 tumors. **b1-b3,** ECDFs of distances from Alt-EJ-like deletion breakpoints to the nearest TSS, shown for individual cancer types within **(b1)** HRD 0-21, **(b2)** HRD 21-42, and **(b3)** HRD >42 tumors. Distances are plotted as log10(distance to nearest TSS + 1 bp). Each colored line represents a cancer type. The vertical dashed line indicates 10 kb from the TSS. Grey shading denotes the ECDF of background deletions (deletions >100 bp for panels a1-a3; deletions <100 bp for panels b1-b3 aggregated across all tumors and HRD groups, providing a reference distribution for comparison.

Cancer	HRD bin	Number of SSA events	Number of SSA events <250 bp from a TSS	Proportion of SSA events <250 bp from a TSS	Number of tumors with ≥1 SSA event
**Biliary-AdenoCA**	HRD 0-21	3	0	0.00	2
**Bone-Osteosarc**	HRD 0-21	1	0	0.00	1
**Breast-AdenoCA**	HRD 0-21	4	0	0.00	3
**CNS-Medullo**	HRD 0-21	4	1	0.25	4
**CNS-PiloAstro**	HRD 0-21	2	1	0.50	2
**Eso-AdenoCA**	HRD 0-21	8	0	0.00	6
**Head-SCC**	HRD 0-21	8	0	0.00	7
**Kidney-RCC**	HRD 0-21	6	0	0.00	6
**Liver-HCC**	HRD 0-21	41	3	0.07	31
**Lymph-BNHL**	HRD 0-21	15	5	0.33	13
**Lymph-CLL**	HRD 0-21	6	3	0.50	6
**Ovary-AdenoCA**	HRD 0-21	2	0	0.00	2
**Panc-AdenoCA**	HRD 0-21	10	0	0.00	10
**Panc-Endocrine**	HRD 0-21	2	0	0.00	2
**Prost-AdenoCA**	HRD 0-21	38	0	0.00	29
**Skin-Melanoma**	HRD 0-21	12	1	0.08	10
**Biliary-AdenoCA**	HRD 21-42	3	0	0.00	2
**Bone-Osteosarc**	HRD 21-42	4	1	0.25	3
**Breast-AdenoCA**	HRD 21-42	19	1	0.05	13
**CNS-Medullo**	HRD 21-42	1	0	0.00	1
**CNS-PiloAstro**	HRD 21-42	0	0	0.00	0
**Eso-AdenoCA**	HRD 21-42	37	1	0.03	22
**Head-SCC**	HRD 21-42	13	0	0.00	7
**Kidney-RCC**	HRD 21-42	4	0	0.00	4
**Liver-HCC**	HRD 21-42	30	1	0.03	22

**Lymph-BNHL**	HRD 21-42	14	1	0.07	6
**Lymph-CLL**	HRD 21-42	0	0	0.00	0
**Ovary-AdenoCA**	HRD 21-42	24	1	0.04	14
**Panc-AdenoCA**	HRD 21-42	34	0	0.00	25
**Panc-Endocrine**	HRD 21-42	1	0	0.00	1
**Prost-AdenoCA**	HRD 21-42	2	0	0.00	2
**Skin-Melanoma**	HRD 21-42	12	0	0.00	9
**Biliary-AdenoCA**	HRD > 42	2	0	0.00	1
**Bone-Osteosarc**	HRD > 42	11	1	0.09	7
**Breast-AdenoCA**	HRD > 42	170	7	0.04	59
**CNS-Medullo**	HRD > 42	0	0	0.00	0
**CNS-PiloAstro**	HRD > 42	0	0	0.00	0
**Eso-AdenoCA**	HRD > 42	29	1	0.03	13
**Head-SCC**	HRD > 42	1	0	0.00	1
**Kidney-RCC**	HRD > 42	0	0	0.00	0
**Liver-HCC**	HRD > 42	11	1	0.09	8
**Lymph-BNHL**	HRD > 42	1	0	0.00	1
**Lymph-CLL**	HRD > 42	0	0	0.00	0
**Ovary-AdenoCA**	HRD > 42	132	2	0.02	43
**Panc-AdenoCA**	HRD > 42	31	0	0.00	17
**Panc-Endocrine**	HRD > 42	0	0	0.00	0
**Prost-AdenoCA**	HRD > 42	18	0	0.00	8
**Skin-Melanoma**	HRD > 42	9	0	0.00	7

Table S1 | Frequency of SSA-like deletions proximal to transcription start sites across cancer types and HRD bin.

Number and proportion of SSA-like deletion events occurring within 250 bp of the nearest transcription start site (TSS) are shown for each cancer type and stratified by homologous recombination deficiency (HRD) bin (0-21, 21-42, >42). For each group, we report the total number of SSA events, the number and proportion occurring within ±250 bp of a TSS, and the number of tumors harboring at least one SSA event. The ±250 bp window corresponds to the central TSS-proximal region highlighted in Fig. S4. Across tumor types, TSS-proximal SSA events are most enriched in select HR-proficient cohorts (HRD 0-21), including lymphoid malignancies (Lymph-BNHL and Lymph-CLL).
